# A Nationwide Analysis of the Phenotype/Genotype Landscape of Hemophagocytic Lymphohistiocytosis: UNC13D Associates with Poor Prognosis

**DOI:** 10.3390/genes16111315

**Published:** 2025-11-02

**Authors:** Dafna Brik Simon, Yarden Greental Ness, Orly Dgany, Sharon Noy-Lotan, Tanya Krasnov, Galit Berger, Tamar Feuerstein, Jerry Stein, Aviva Kraus, Asaf Yanir, Assaf Barg, Elad Jacoby, Noa Mandel-Shorer, Dan Harlev, Ehud Even-Or, Hannah Tamary, Oded Gilad, Orna Steinberg-Shemer, Joanne Yacobovich

**Affiliations:** 1 Department of Pediatric Hematology Oncology, Schneider Children’s Medical Center of Israel, Petah Tikva 4920235, Israel; dafna.briksimon@sickkids.ca (D.B.S.); galitpi@clalit.org.il (G.B.); tamarpo1@clalit.org.il (T.F.); jstein@clalit.org.il (J.S.); asafdavidya@clalit.org.il (A.Y.); odedgi@clalit.org.il (O.G.); ornas2@clalit.org.il (O.S.-S.); 2 Gray Faculty of Medical and Health Sciences, Tel Aviv University, Tel Aviv 39040, Israel; assaf.barg@sheba.health.gov.il (A.B.); elad.jacoby@sheba.health.gov.il (E.J.); htamary@post.tau.ac.il (H.T.); 3 Pediatrics Department, The Edmond and Lily Safra Children’s Hospital, Sheba Medical Center, Ramat Gan 52621, Israel; yarden.greentalness@sheba.health.gov.il; 4 Felsenstein Medical Research Center, Petah Tikva 4920235, Israeltanyak@clalit.org.il (T.K.); 5 Hematology-Oncology Division Safra Children Hospital, Sheba Medical Center, Ramat-Gan 52621, Israel; 6 Department of Pediatric Hematology-Oncology, Ruth Rappaport Children’s Hospital, Rambam Medical Center, Haifa 3109601, Israel; n_mandel@rambam.health.gov.il; 7 The Dyna & Fala Weinstock Department of Pediatric Hematology Oncology, Faculty of Medicine, Hebrew University of Jerusalem, Hadassah Medical Center, Jerusalem 91120, Israel; danha@hadassah.org.il; 8 Department of Bone Marrow Transplantation and Cancer Immunotherapy, Jerusalem 91120, Israel; evenor@hadassah.org.il; 9 Faculty of Medicine, Hebrew University of Jerusalem, Jerusalem 9190500, Israel

**Keywords:** hemophagocytic lymphohistiocytosis, familial HLH, genotype/phenotype association, germline disease

## Abstract

Background/objectives: Geographic and ethnic differences influence the genetic landscape of hemophagocytic lymphohistiocytosis (HLH) and the frequency of familial HLH (FHL); this in turn can affect outcomes. Methods: We collected data on 98 patients treated for HLH between 1 January 2001 and 31 July 2024 at four tertiary centers, characterizing the genotype/phenotype correlations. Results: Half of the patients, 51 (52%), were symptomatic by age 1 year and 43 (44%) were diagnosed by that age. Our varied population included 43% Sephardic/Ashkenazi/Ethiopian Jews, 50% Muslim Arabs, and 7% Druze. Molecular analysis was performed on 90.5% of patients and revealed an FHL-related variant in 72%. The genetic variation included biallelic variants in *PRF1* (21), *UNC13D* (12), *STXBP2* (15), and *STX* (1). Eight hemizygous variants were found in X-linked lymphoproliferative disorder-related genes. A *RAB27A* monoallelic variant in an infant with a severe phenotype was considered pathogenic. The recently described HLH-related gene, *ZNFX1*, was mutated with varying penetrance in three symptomatic siblings. Overall, of the 94/98 with follow-up, 77% are alive. Strikingly, 5/12 (41.6%) patients with *UNC13D* variants died while 14/15 (93.3%) patients with *STXBP2* variants survived. Logistic regression found poor prognosis associated with young age at diagnosis (*p* < 0.001), any variant (*p* = 0.016), *UNC13D* variant (*p* < 0.001), poor initial treatment response (*p* = 0.009), and no BMT (*p* = 0.005). Conclusions: Our cohort included an extremely high rate of genetic testing and detection of FHL-related variants. *UNC13D* variations are associated with exceedingly poor outcomes. Response to initial treatment seems crucial for positive outcomes, as does access to hematopoietic stem cell transplantation. Overall, we report a high survival rate, possibly due to a high index of suspicion and prompt diagnosis.

## 1. Introduction

Hemophagocytic lymphohistiocytosis (HLH) is a life-threatening immuno-hematological syndrome displaying uncontrolled hyperinflammation due to the hyperactivation of the T lymphocytes and NK cells in response to a pathological stimulus, most commonly an infectious agent [1,2]. The diagnosis of HLH is traditionally based upon diagnostic criteria (Table S1). Clinical manifestations include persistent high fever, hepatosplenomegaly, cytopenias, edema, rash, and possible central nervous system involvement. Characteristic laboratory findings include pancytopenia, extreme elevation of ferritin, triglycerides, and liver function tests, along with decreased fibrinogen. The typical immunological impairment manifests as elevated soluble IL-2r (CD25) and decreased NK cell activity [1,3].

Historically, the disease is classified into primary/familial HLH (FHL), caused by genetic defects, or secondary HLH. More recently, Jordan et al. suggested the concept of “HLH disease mimics” instead of “secondary HLH” to emphasize the various specific etiologic associations other than genetic causes [3].

In infants and young children, the clinical picture is predominantly caused by pathogenic variants in genes responsible for the activation and proliferation of NK cells and cytotoxic T lymphocytes [2]. Classic familial HLH (FHL) is primarily caused by autosomal recessive defects in the *PRF1*, *UNC13D*, *STX11*, and *STXBP2* genes encoding for proteins that are involved in perforin-mediated cytotoxic activity [4]. Additional syndromes characterized by hyperinflammation are due to loss-of-function variants in the *LYST*, *RAB27A*, and *AP3B1* genes leading to interference in the formation or transport of cytotoxic granules [4]. Chediak–Higashi syndrome, Griscelli syndrome type 2, and Hermansky–Pudlak syndrome type 2 present with similar HLH clinical manifestations [5,6,7].

Additionally, certain genetic disorders associated with immune deficiency are at high risk of developing HLH. X-linked lymphoproliferative syndrome types 1 and 2 (XLP1 and XLP2), caused by variants in the *SH2D1A* [8] and *XIAP* [9] genes, respectively, present with hyperinflammation often related to EBV antigenemia. ZNFX1 deficiency predisposes to severe viral infections and an HLH-like multisystem inflammatory disease as a result of dysbalanced interferon production [10]. CD27 deficiency [11] and interleukin-2 inducible T-cell kinase (ITK) deficiency [12] are also associated with the HLH phenotype.

The effect of geographic and ethnic variability on the prevalence of the various genetic causes of FHL is an important factor in the evaluation of patients. Studies have shown the significant genetic diversity among populations and the associated influence on appropriate therapy and outcome [13,14,15,16,17].

Acquired or secondary HLH can manifest at any age and is overall more common than genetic HLH. It is usually a hyperinflammatory syndrome due to infections; mostly Herpes family viruses [18], malignancies, autoimmune/rheumatic diseases, metabolic disorders, biological/cellular therapies, and/or acquired immunodeficiency disorders.

Diagnosis of the syndrome requires a high index of suspicion. The timely diagnosis is critical for initiating prompt, effective treatment which has been proven to affect survival. As there is no single pathognomonic finding for HLH, the combination of prolonged fever, hepatosplenomegaly, and pancytopenia should trigger specific diagnostic testing [19]. To date, the diagnostic criteria initially suggested in the HLH-94 study protocol and then refined in the HLH-2004 study remain the mainstay [20]. Often, in the early stages of the disease, not all criteria are evident; rather, they accumulate over the disease course.

Without treatment, FHL is a terminal disease. The study-based treatment protocols include a combination of immunosuppressive medications (glucocorticosteroids) with cytotoxic therapy (etoposide). Cyclosporine A was a part of both HLH-94 and HLH-2004 study protocols; however, more recent results negated any advantage to its use [21,22].

Currently, treatment options for relapsed/refractory primary HLH also include emapalumab, an FDA-approved monoclonal antibody binding interferon gamma, and JAK inhibitors are under investigation [23,24]. Allogeneic hematopoietic stem cell transplantation (HSCT) is the only curative therapy in FHL; without HSCT, the long-term outcome is fatal. In this study, we aimed to analyze the genetic variants, genotype–phenotype findings/associations, and outcomes of 98 patients with HLH treated at four tertiary centers in Israel.

## 2. Materials and Methods

### 2.1. Study Population

This retrospective cohort study included pediatric patients with HLH diagnosed between1 January 2001 and 31 July 2024. They ranged in age from one week to 17 years and were diagnosed at different medical centers in Israel: Hadassah-Hebrew University Medical Center, Sheba Medical Center, Rambam Medical Center, and Schneider Children’s Medical Center. These patients underwent molecular evaluation for HLH at the Felsenstein Molecular Hematology Laboratory at Schneider Children’s Medical Center or through whole-exome sequencing. Inclusion criteria included patients who (1) met the diagnostic criteria for HLH [Table S1]; (2) received treatment according to clinical protocols; and (3) had follow-up data available, enabling evaluation of outcomes. Exclusion criteria included incomplete diagnostic workups and evaluations performed without clinical disease (e.g., prenatal diagnosis). This study was approved by the ethical review board at each participating medical center.

### 2.2. Data Collection

Patient data were extracted from medical records and included age, gender, gestational week and birth weight, developmental history, parental ethnicity, presence or absence of consanguinity, clinical presentation, age at symptom onset and diagnosis, laboratory findings (ferritin [Simens ADVIA Centaur XP and ADVIA Centaur XPT Systems 10629858], PT derived fibrinogen [*ACL-TOP 550* System with Recomboplastin 2G; Instrumentation Laboratory: Bedford, MA, USA], NK cell function [25], soluble IL2R (CD25) [Human sIL-2R ELISA kit. Invitrogen REF BMS212-1; ThermoFischer Scientific: Waltham, MA, USA], flow cytometry for CD 107a [26] and perforin [27], white blood cell count, neutrophil count, hemoglobin, platelets, bone marrow, and CSF results), genetic testing, imaging, treatment protocol, additional immunosuppressive therapies, treatment response, necessity and response to HSCT, and mortality (Table S2).

#### 2.2.1. Specialized Hematologic Tests

- NK Cytolytic Function Assay: NK cell-mediated lysis of K562-labeled cells was assessed using a CR51-release assay [25].
- Soluble IL2R: The quantitative determination of soluble interleukin-2 receptor (sIL2R) in serum was performed using a sandwich enzyme-linked immunosorbent assay (ELISA) kit according to the manufacturer’s instructions. Serum samples were analyzed alongside calibrated standards to establish a standard curve, and sIL2R concentrations were calculated by interpolation. The assay demonstrated an analytical level of sIL2R in U/mL unit.
- Flow cytometry:
  o Degranulation assays quantifying CD107a surface expression were performed in a small subset of patients to direct molecular analyses for FHL [26].
  o Samples were analyzed using a FACSCalibur flow cytometer (Becton Dickinson, San Jose, CA, USA) in a small subset of patients to correlate with molecular findings [27].

#### 2.2.2. Genetic Diagnosis

DNA and cDNA extraction, amplification, and sequencing were conducted using standard laboratory techniques. In early years, patients’ samples were tested using Sanger sequencing for the suspect genes as they were identified, including *PRF1*, *UNC13D, STXBP2, STX11, SH2D1A*, *XIAP,* and *ITK*.

From 2016, patients underwent molecular assessment either by NGS panel in the Molecular Hematology Lab at the Felsenstein Research Center or by experimental whole-exome sequencing.

Our custom-made NGS panel is continuously updated; in its original format, immunologic diseases and rare anemias were separated from a panel for bone marrow failure and cancer predisposition. In 2022, the 2 panels were combined; the more recent version includes 491 known genes that cause hematologic diseases, including those with immunologic manifestations (Table S3). Panel design, library preparation, and sequencing procedure were performed as previously described [28]. Since 2016, Sanger sequencing was mainly used to confirm NGS findings and to study family segregation.

##### Sequencing Results and Bioinformatics Pipeline

Sequencing reads were aligned against a reference genome (GRCh37/UCSC hg19) and variants were identified and labeled using the SureCall software (v.3.5.1.46; Agilent Technologies, Santa Clara, CA, USA). Single nucleotide polymorphism filtering was established as previously described [28], using both an in-house platform and the Emedgene AI-based genomic analysis platform (Emedgene Technologies, Tel-Aviv, Israel). Genetic variants were reported in keeping with the American College of Medical Genetics guidelines [29,30]. In general, only pathogenic and likely pathogenic results were reported. However, in cases of homozygous VUS, with strong clinical suspicion of FHL, in the absence of another initiating factor, we considered the variants as most probably the pathogenic cause of the disease.

##### Copy Number Variants Detection

Copy number variant (CNV) analysis was performed using the “Rainbow”—Genoox CNV Caller. Rainbow Caller employs a machine learning-based anomaly detection algorithm, in which variants are determined based on exon-level coverage, using a cohort of samples (*n* > 30). The model considers multiple factors including GC content, coverage variance over different samples, and neighboring gene coverage (Genoox, Tel-Aviv, Israel). CNV findings were confirmed by multiplex ligation-dependent probe amplification (MLPA, MRC, Amsterdam, Holland) [31].

### 2.3. Statistical Analysis

Data were collected using Microsoft Excel, and statistical analyses were performed using IBM SPSS Statistics Version 29.0.1.0 (171). Frequencies were calculated using mean values ± standard deviation, or medians and range for continuous variables and percentages for nominal or ordinal descriptives. Chi-square tests and the *t*-test were used for univariate analysis, and logistic regression and ANOVA were employed for multivariate analysis. A *p*-value of <0.05 was considered statistically significant.

## 3. Results

### 3.1. Study Cohort

Our study included 98 pediatric patients treated between 1 January 2001 and 31 July 2024 at four tertiary centers in Israel (Table 1). A total of 40 children (41%) were treated at the Schneider Children’s Medical Center of Israel, 28 (29%) at Sheba Medical Center, and the remaining divided between Hadassah Medical Center and Rambam Medical Center. These centers represent the four major pediatric hematology-oncology departments providing the majority of pediatric hematopoietic stem cell transplant services in Israel. The median ages at onset of symptoms and diagnosis were close: 12 months (0.1–204 months) and 15 months (0.3–205 months), respectively. Fifty-one patients (52%) exhibited symptoms before the age of 1, while in 63 patients (66%) clinical onset was before 24 months of age. Diagnosis (clinical or molecular) was reached before the age of 1 in 45%. Males represented 55% of the study population.

Our study population included 43% Jews of Sephardic, Ashkenazi, and Ethiopian origin, 50% Muslim Arabs, and 7% Druze. Parental consanguinity was reported in 39 (44%) of cases for which these data were available, exclusively among Muslim and Druze patients. In 19 cases (18%) there was a positive family history of HLH.

### 3.2. Clinical Presentation

**Figure 1:**
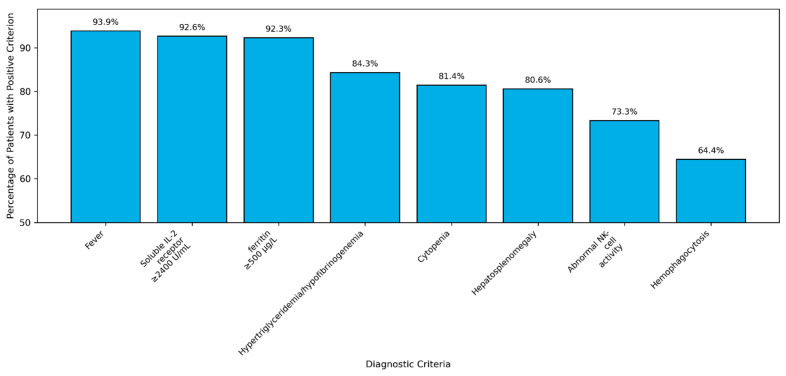
Diagnostic criteria at the time of diagnosis. Legend: Hypertriglyceridemia = ≥265 mg/dL, hypofibrinogenemia = ≤150 mg/dL, cytopenia = greater than 2 standard deviations below normal for age, abnormal NK activity = <20%.

HLH-2004 diagnostic criteria (Table S1) were fulfilled in over 60% of cases (Figure 1).

Fever (92; 94%), hepatosplenomegaly (79; 81%), and cytopenias (79; 81%) were the most common presenting signs and symptoms in our study cohort. Central nervous system involvement at onset was noted in 30 (31%) of patients. Less common symptoms were adenopathy (24; 24%), rash (25; 25.6%), and anasarca (30; 30.6%). Ferritin was above the diagnostic level of 500 ng/L in 58 patients and above 1000 ng/L in 47 (81% of those with high ferritin). When more specific laboratory testing was performed, CD25 (Soluble IL2 receptor ≥ 2400 U/mL) was above the diagnostic cut-off value in 64/69 (92%) and NK activity was below the threshold (<20%) in 38/52 of patients tested (73%). Bone marrow hemophagocytosis was found in 58/90 cases (64.4%).

### 3.3. Genetics

In 81 (82.7%) cases, molecular analysis was performed to assess a genetic cause for the disease; 78% of Jewish patients and 84% of the Muslim patients were evaluated. Disease-causing biallelic/hemizygous variants were detected in 61 (75%) of those tested; 37 (74%) were found in Muslims while 16 (38%) in Jewish patients. A genetic cause was found in the majority of Muslim patients (88%), while only 48% of Jewish patients were diagnosed with a genetic cause of their disease.

Regarding time of onset and diagnosis, the median age at symptom onset was 9 (0.1–132) months in cases with a genetic diagnosis, compared to 14 (0.3–204) months in patients without (*p* < 0.001). Similarly, the median age at diagnosis for patients with a genetic cause for HLH was significantly younger than that for children without (11 {0.1–180} months vs. 15 {1–205}, *p* = 0.003).

**Figure 2:**
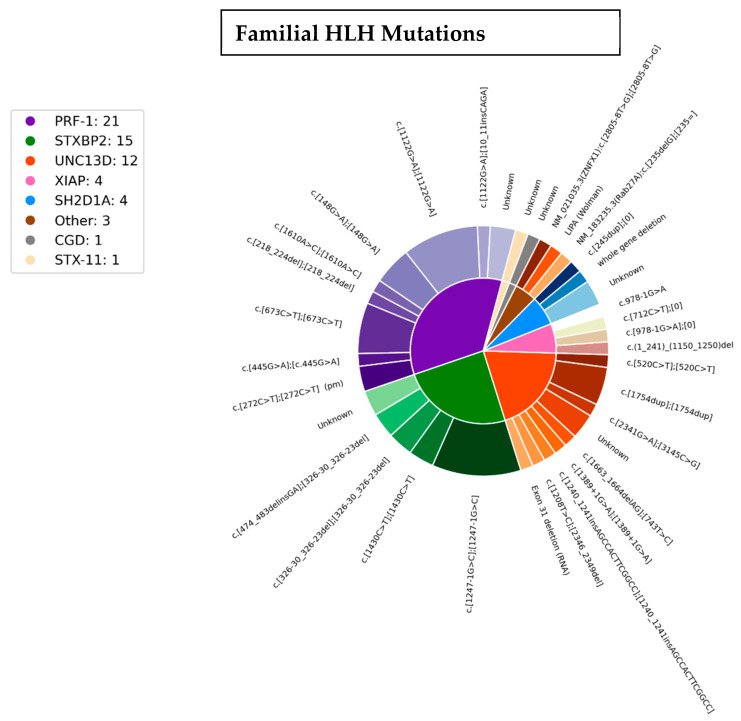
The genetic diversity of disease-causing variants in FHL. Legend: Other = one case each of RAB27a, Wollman’s disease, ZNFX1; CGD = chronic granulomatous disease.

The genetic diversity of disease-causing FHL- (*PRF1* {FHL-2}, *UNC13D* {FHL-3}, *STXBP2* {FHL-5}) and HLH-related genes (*SH2D1A*, *XIAP*, *RAB27a*, *ZNFX1*), as well as clinically diagnosed diseases such as chronic granulomatous disease (CGD) and Wolman’s disease, in our cohort is depicted in Figure 2. *PRF1* was the most commonly mutated gene (twenty homozygotes and one compound heterozygote), *STXBP2* was bi-allelically mutated in 15 patients, two of which were compound heterozygous, and *UNC13D* was pathogenically mutated in 12 patients, of which eight were homozygotes and four compound heterozygotes. Additionally, in three patients only one variant of *UNC13D* was found. One patient was simultaneously a homozygote for the NM_001083116.3(*PRF1*):c.272C>T (p.Ala91Val) and a compound heterozygote for two-point variants, c.2341G>A and c.3145C>G in *UNC13D*. Only one patient in our study was diagnosed with bi-allelic variants in *STX11* {FHL-4}.

In addition to the classic FHL genes, many additional HLH-associated genes were found to be variant in our patient population. X-linked lymphoproliferative disease (XLP) was diagnosed in eight patients: four cases each of *SH2D1A* (XLP1) and *XIAP* (XLP2) variants. One patient was diagnosed with a homozygous variant in the newly described *ZNFX1* gene. One patient each was diagnosed with Wolman’s disease and chronic granulomatous disease (CGD). An additional patient was diagnosed with clinical FHL (repeated episodes until full-blown life-threatening HLH) however only one deletion was diagnosed in the gene for Griscelli syndrome type 2 (*RAB27A*).

**Figure 3:**
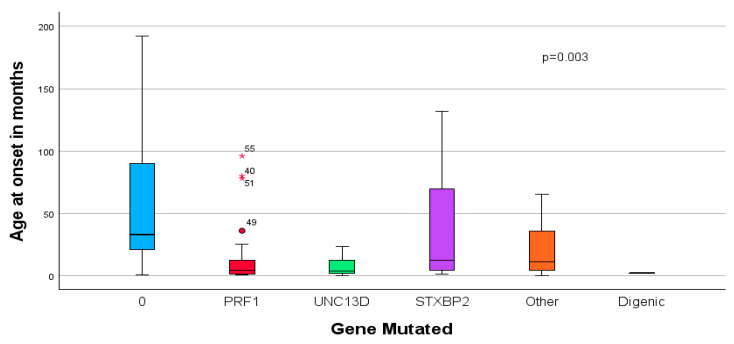
Genetic variation in HLH age at onset. Legend: Other: XIAP = 4, SHD2A = 2, RAB27A = 1, clinical case of chronic grantulomatous disease = 1, clinical case of Wolmans’ Disease = 1, Digenic: PRF1 homozygote + UNC13D compound heterozygote Dotted line represents the median age at symptom onset.

Using ANOVA analysis, we found that the mean age at onset and diagnosis was significantly younger among patients with *UNC13D* variants when compared to all other variants or no variant found (6.7 ± 3.5 months vs. 29.88 ± 22.1 vs. 59 ± 29, *p* = 0.003; 13.4 ± 9.5 months vs. 40.5 ± 26 vs. 67.6 ± 29.1 months, *p* = 0.009, respectively) (Figure 3).

### 3.4. Outcome Prediction

Univariate analysis revealed a statistically significant relationship between consanguinity (*p* = 0.045), response to therapy (*p* < 0.001), treatment with HSCT (*p* = 0.012), diagnosis of any genetic cause (*p* < 0.001), and specific genetic causes (*p* = 0.059) and outcomes. Of the 23 deaths, 6 patients had not undergone genetic testing, in 4 genetic testing did not reveal a pathogenic variation, and in the remaining 13 (56%) cases a genetic variant was identified. A total of 78% (48/61) of patients with a genetic diagnosis were alive at the time of this summary, compared to 100% (20/20) of those without a genetic diagnosis (assumed secondary HLH) being alive. There was a clear association between particular genetic diagnoses and outcomes; specifically, 5/12 (41.6%) patients with *UNC13D* variants were non-survivors while only 1/15 (6.6%) patients with *STXBP2* variants did not survive (*p* < 0.001).

No statistical significance was found in the univariate analysis between outcome and ethnicity; positive family history; presenting symptoms including fever, HSM, adenopathy, rash, and anasarca; general laboratory values at diagnosis; elevated levels of Sol IL2r; low NK activity; ferritin; or HLH treatment protocol used.

### 3.5. Logistic Regression

Logistic regression found that poor prognosis was associated with young age at diagnosis (*p* < 0.001), any variant found (*p* = 0.016), variant in *UNC13D* (*p* < 0.001), poor response to initial treatment (*p* = 0.009), and no BMT (*p* = 0.005).

The other clinical parameters were not found to predict outcome in this analysis.

## 4. Discussion

In this study, we collected demographic, clinical, and molecular data from 98 patients diagnosed and treated for HLH in Israel during a period of over 23 years and analyzed genotype–phenotype correlations. All patients included fulfilled HLH-2004 criteria and were treated in one of the four tertiary pediatric centers in the country. Over half of the patients developed symptoms before one year of age, and two-thirds presented with typical features by age two. The time lag between symptom onset to diagnosis was short, suggesting a high index of suspicion among treating pediatric hematologists. Per inclusion criteria, the clinical picture of our cohort fulfilled the accepted diagnostic criteria in children; notably, all eight of the diagnostic criteria occurred in over 50% of patients. Since 19/98 (19.4%) patients had a positive family history, presumably a few patients were diagnosed due to a known familiar molecular diagnosis pre-empting the full clinical picture; however, this information was not specifically collected.

Overall, the study population represented the ethnic variety of the country. However, due to high rates of consanguinity in the Arab population and the common autosomal recessive inheritance in FHL, Muslims and Druze were over-represented (50% and 7%, respectively) in comparison to the overall Israeli population (73% Jewish, 18% Muslim, 1.6% Druze, Israel Central Bureau of Statistics) [32].

Rigorous molecular diagnosis of our cohort was performed with the goal of maximizing diagnostic potential. A total of 82.7% of patients underwent a molecular work-up and in nearly 75% of them a genetic cause of their disease was identified. The effects of high rates of parental consanguinity are manifest in the 90% rate of genetic diagnoses among Muslim patients, in which consanguinity is prevalent. This is consistent with a recent report from Saudi Arabia (a Muslim country with a high rate of consanguinity) where an FHL or FHL-related gene was identified in 69/87 (79%) of patients tested [33]. Other populations outside of the Middle East show markedly lower rates of FHL. Studies from the United States found that only 10–15% of patients were positive for disease-causing FHL variants [15,16]. In the first study, a pediatric HLH cohort analysis from the United States used whole-exome testing and identified pathogenic variants in only 19% of 101 cases evaluated [16]. The second study of 1892 samples from patients aged 1 day to 78 years, referred to the Cincinnati Children’s Hospital Medical Center Molecular Diagnostic Laboratory and analyzed using targeted NGS panels, found a potentially causal genetic diagnosis in 12%. There was a 40% genetic detection rate in children under the age of 5 years (28.6% among 332 patients under the age of 1 year) while only 6.7% among 6–12-year-olds, 3.3% among 12–18-year-olds, and 4.8% of cases analyzed among adults (over 18 years old) revealed a molecular diagnosis of FHL [15]. Similar results were reported in a Chinese study of 265 HLH patients (aged 1 month–56 years, median 3 years) which found 37 (14%) with disease-causing variants. Notably, only six genes were analyzed in this Chinese study using Sanger sequencing: *PRF1*, *UNC13D*, *STXBP2*, *STX11*, *SH2D1A* and *XIAP* [34]. The Italian registry study reported on 500 cases of HLH among a patient population aged 0–60 years (median 2.2 years) at diagnosis. In their report, 34% of 500 cases were molecularly diagnosed with FHL or FHL-related disease [17].

Unsurprisingly, patients with FHL were symptomatic and diagnosed at a much younger age, supporting the notion that when a genetic cause is present, HLH will usually present within the first years of life [15,16,17].

A wide variety of genetic diagnoses were found in our cohort, including the involvement of 10 different genes. Similarly to reports from Western populations, *PRF1* was the most commonly mutated gene [15,16,17] with *STXBP2* was in second place. Interestingly, in certain Eastern countries, such as Korea and China, *UNC13D* is the most common genetic cause of FHL, whereas in Japan *PRF1* is the most commonly affected gene [34]. *STXBP2* is the second most mutated gene in our population. This gene is the most highly mutated in Saudi Arabia and neighboring Arab countries [33]. The molecular diagnoses in our cohort of *PRF1* and *STXBP2* were mainly represented by homozygous variants while in patients with *UNC13D* variants 1/3 were compound heterozygotes. This observation is in keeping with the gene prevalence of *PRF1* and *STXBP2* among consanguineous populations (enriching homozygous inheritance), while *UNC13D* is a more prevalent cause of FHL in non-consanguineous ethnicities. *STX11* variants as a cause of FHL, which have been primarily described in the Turkish population, were extremely rare in our cohort [14].

### Outcome and Genetic Diagnoses

Most clinical and laboratory data were not found to be related to survival in univariant analysis. In contrast, logistic regression revealed that genetics and therapy are the main predictors for mortality. In particular, genetically based disease was a major predictor for risk of morbidity, as was poor therapy response and accessibility to bone marrow transplantation. Sixty-one percent of the cases of death were in patients with FHL, while in secondary causes of HLH the survival was significantly greater than among cases of FHL.

The relationship between genetic HLH and negative outcome has been described [17]; however, it is understandably less pronounced in populations with lower molecular diagnostic rates. We recently published our findings on the predictive value of early response on improved outcome in FHL [35].

*UNC13D* in our cohort was diagnosed in cases where patients presented at a younger age and was associated with the poorest survival in both univariate analysis and logistic regression. This finding was also suggested by the Saudi study [33] but not described in other studies and warrants further investigation.

Three cases in our cohort had unusual genotype–phenotype features which will be discussed in detail.

One interesting finding is the age difference between two identical twins with a homozygous *STXBP2* variant (NM_006949.4:c.[1247-1G>C];[1247-1G>C]). The index case was diagnosed with FHL at age 5 years 9 months; he received treatment according to the HLH-2004 protocol and was transplanted from a matched cord blood unit. He ultimately succumbed to complications of the transplant. His twin brother was not being followed and arrived at another center at age 11 with full-blown HLH. He was rapidly diagnosed, treated, and underwent prompt HSCT. He is currently healthy at 8 years old post-therapy. The gap in clinical onset among siblings with FHL has been previously described [17] and may be attributed to a particular exposure or triggering agent.

The newly described *ZNFX1* gene related to FHL and its tendency for viral infections was described in three cases in one family. The first case was a 5-month-old girl diagnosed with severe HLH in 2007; she had poor response to the HLH-2004 protocol, received alemtuzumab (anti-CD 52) as salvage therapy, and eventually died. Molecular work-up at the time revealed only a monoallelic PRF1 A91V variant (debatably a polymorphism or mildly pathogenic). Seven years later, her sister was followed for mild–moderate thrombocytopenia and increased liver function tests (LFTs) during febrile illnesses without ever developing into full-blown HLH. Molecular testing by our NGS-targeted panel did not reveal an HLH-related variant and eventually she was lost to follow-up. In 2024, a 6-month-old sister was seen in our clinic for mild thrombocytopenia and increased LFTs; again, no HLH variant was found in our NGS panel. At age 9 months she was admitted to another center with necrotizing encephalitis. Whole genome work-up revealed a homozygous variant in the *ZNFX1* gene, which was first described just months after the last update to our NGS panel. Neither of the two older sisters have undergone HSCT to date. The specific clinical data for the two older sisters are not included in this study due to parental non-consent beyond the case description. Obviously, there is variable penetrance in the clinical manifestations of this disease.

A third case of note is the clinically severe HLH in a male patient with a monoallelic pathogenic variant in the *RAB27A* gene. The diagnosis was made using our targeted panel after he fully fulfilled the HLH-2004 criteria, including ferritin levels of 100,000 mcg/L Further analysis was warranted due to the extreme phenotype; however, WES trio analysis did not contribute to the diagnosis. The mother was found to be a carrier of the same variant while the father was wild-type for all known HLH-related genes. Surprisingly, CD 107a expression was abnormal in the patient’s father and paternal grandmother; however, no other molecular findings were recognized. The patient underwent allogeneic HSCT, suffered from severe graft versus host disease, and eventually died of overwhelming fungal sepsis. The HLH phenotype has been described in two cases with monoallelic variations in *RAB27A* that were different from the one found in our patient, however in close chromosomal proximity [36].

The weaknesses of this study stem from the retrospective nature of data collection leading to reporting bias, the varying treatment protocols over the study period, and currently accepted treatment practices which are not related to a study protocol. Additionally, molecular testing has advanced since the early patient analyses possibly, leading to missed molecular diagnoses.

## 5. Conclusions

Our study emphasized that the genetic variability of FHL is largely dependent on the ethnic background of individual populations and specifically the rate of consanguinity. FHL manifests at a younger age in our population, especially in patients with *UNC13D* variants. This study demonstrates that outcomes in HLH are influenced by treatment response, genetic background, and the availability of allogeneic HSCT. Notably, *UNC13D* mutations were associated with particularly poor prognosis; due to the small sample size and in light of the paucity of reports in the literature concerning outcomes in this subset, it would be prudent to collaborate data on a larger cohort to explore this genotype–phenotype association. Recognition of all these factors is essential for guiding therapeutic strategies and improving management of patients with HLH.

## Figures and Tables

**Table 1:** Patient Characteristics.

	Total	Percent (%)
Age at onset ≤ 1 yr	54/95	55
Age at diagnosis (months)		
≤12	43/96	45
>12	53/96	55
Gender (M)	54	55
Ethnicity		
Muslim	50	51
Jewish	42	43
Druze	6	6
Consanguinity		
Muslim	36	39.5
Jewish	0	
Druze	4	4.5
Total	40/91	44
Presenting clinical manifestations		
Fever	92	94
Hepatosplenomegaly	79	81
Adenopathy	24	24
Rash	25	25
CNS involvement	30	31
Edema/anasarca	30	31
Presenting laboratory manifestations		
Cytopenias	79/95	81
Triglycerides ≥ 265 mg/dL	65/83	78
Fibrinogen ≤ 150 mg/dL	68/83	82
Ferritin ≥ 500 μg/L	85/92	92
NK activity < 20%	38/52	73
Soluble IL2 receptor ≥ 2400 U/mL	64/69	92
Treatment		
HLH-94	12	12
HLH-2004	62	63
Steroids/IVIG	21	21
Other/Second-line therapy	22	22
Bone marrow transplantation	58	59

## Data Availability

Data supporting this study are not publicly available due to ethical considerations.

## Supplementary Materials

The following supporting information can be downloaded at https://www.mdpi.com/article/10.3390/genes16111315/s1, Table S1: HLH-2004 Diagnostic Criteria, Table S2: Patient’s characteristics, Table S3: NGS Panel gene list 2025.
