# Supplementary material for: A Nationwide Analysis of the Phenotype/Genotype Landscape of Hemophagocytic Lymphohistiocytosis: UNC13D Associates with Poor Prognosis
Source: Genes (Basel). 2025 Nov 2;16(11):1315. doi: 10.3390/genes16111315 (PMC12652584; doi:10.3390/genes16111315)
Supplement: Supplementary file 1 [file genes-16-01315-s001.zip › genes-3861651-supplementary.pdf]

Pt	Age at onset (month)	Age at diagnosis (month)	Sex	Ethnicity	Consan-guinity	Clinical manifestation (HLH criteria met)	Hb (g/dL)	PLT (100 × 10^{9}/L)	ANC (0.10* 10^{9}/L)	TG (mg/dL)	Fib. (mg/dL)	Ferritin (µg/L)	Hemophago-cytosis in BM	Flow cytometry	NK Activity (%)	sIL-2R (U/ml)	Genetic diagnostic method	Gene	Variant	Tx.	Outcome
1	25	25	F	1	Yes	Fever, HSM, EBV + (7/8)	8.9	81	1300	438	93	2247	Yes	1,4	N	7795	NGS panel	N	-	Mabthera + Dexa	CR
2	2	2	F	1	Yes	Fever, Elevated LFT, Edema (5/8) Wolman Disease	6	69	900	356	116	70,000	No	N/A	N/A	High	WES	Wolman	-	Dexa	CR
3	12	12	M	1	Yes	Fever, HSM, Rash, CNS involvement, EBV+ (6/8)	6.7	8	250	N/A	360	17,742	Yes	2	21%	13,000	NGS panel	UNC13D	NM_199242.3:c.[2179C>T]; c.[2179C>T]	2004 Protocol Mabthera BMT	CR
4	38	38	M	1	No	Fever, HSM, Rash, Encephalopathy (6/8)	9	127	780	425	74	1443	No	N/A	N/A	12,000	NGS panel	SH2D1A	-	2004 Protocol Mabthera BMT	CR
5	11	11	M	1	Yes	Fever, HSM, CNS involvement (8/8)	6.7	312	N/A	423	119	6200	Yes	N/A	18%	High	WES	SH2D1A	-	2004 Protocol	deceased
6	24	24	M	2/3	No	Fever, HSM (7/8)	6.9	77	680	161	47	4400	Yes	N/A	N/A	7400	PRF1, STX, UNC13D, STXBP2	N	-	2004 Protocol	CR
7	192	192	F	3	No	Fever, HSM, Edema (6/8)	7.9	98	100	199	86	1500	No	N/A	N	12,036	N/D	-	-	2004 Protocol	CR
8	14	15	M	1	N/A	Fever, Adenopathy, EBV+ (5/8)	10.9	164	400	460	91	1800	Yes	N/A	N/A	2400	PRF1, STX, UNC13D, STXBP2, SH2D1A, XIAP	SH2D1A	NM_002351.5(SH2D1A): c.[245dup]	2004 Protocol Mabthera	deceased
9	65	72	M	1	No	Fever, HSM, Adenopathy, Edema (8/8)	6.5	75	500	492	141	15,000	Yes	N/A	5.8%	1094	WES	XIAP	NM_001167.4(XIAP):c.[712C>T];[0]	2004 Protocol+ BMT	CR
10	56.5	56.5	M	2	No	Fever, HSM, Rash, CMV+ (5/8)	8.6	81	680	216	256	39,607	Yes	1	N	2183	NGS panel	XIAP	NM_001167.3(XIAP):c.[978-1G>A];[0]	Dexa Gancyclovir	CR
11	4	5	M	2	No	Fever, HSM (6/8)	6.6	58	740	504	118	17,154	no	N/A	N/A	14,100	PRF1, STX, UNC13D, STXBP2 Rab27a, SH2D1A, XIAP	UNC13D	NM_199242.3:c.[2782C>T[;c.[2782C>T]	2004 Protocol BMT	CR
12	9	10	M	1	Yes	Fever, HSM, Adenopathy (5/8)	7	10	0	200	153	15,000	Yes	N/A	N/A	N/A	N/D	-	-	2004 Protocol	deceased
13	9	9	M	1	N/A	Fever, HSM, Adenopathy, CNS involvement, Edema (6/8)	6.3	4	300	505	84	15,000	Yes	N/A	N/A	N/A	N/D	-	-	2004 Protocol	deceased
14	36	36	M	1	Yes	Fever, HSM, Rash (6/8)	7	14	150	366	120	5025	Yes	N/A	N/A	N/A	NGS panel	SH2D1A	-	Prednisone+ IVIG+BMT	CR
15	18	19	F	1	Yes	Fever, HSM, Rash, CNS involvement, CGD (7/8)	6	40	4000	188	750	944	Yes	N/A	<20%	2583	WES	CGD	-	2004 Protocol	N/A
16	8	10	M	2	No	Fever, Rash (6/8)	7	100	2600	372	124	5000	Yes	2	N	6100	NGS panel	RAB27A	-	HLH-94 BMT	deceased
17	5	5.5	F	1	Yes	Fever, HSM, Rash (8/8)	9.7	9	1000	430	414	2500	Yes	N/A	0%	3387	PRF1, STX, UNC13D	PRF1 ZNFX1	NM_001083116.3:c.[272C>T]; [272C>T] NM_021035.3(ZNFX1):c.[2805-8T>G];[2805-8T>G]	2004 Protocol Alemtuzumab	deceased
18	0	1	F	2	No	Fever, HSM, CNS involvement, Edema (6/8)	8.9	491	220	333	150	2346	Yes	N/A	N/A	N/A	PRF1, STX, UNC13D, STXBP2,Rab27a,ITK	N	-	2004 Protocol ATG	deceased
19	39	75	M	1	Yes	fever, HSM, Adenopathy, CNS involvement, EBV+ (8/8)	7.5	10	100	499	N/A	2620	Yes	N/A	<20%	48,205	PRF1	N	-	2004 Protocol Mabthera, IVIG BMT	CR
20	1	3	F	3	No	fever, HSM, CNS involvement (5/8)	4.3	60	300	N/A	N/A	10,600	Yes	N/A	N/A	N/A	PRF1, STX, UNC13D, STXBP2,RAB27a,ITK	UNC13D	NM_199242.3:c.[1208T>C];[2346_2349del]	2004 Protocol BMT	CR
21	36	96	F	1	Yes	Ffever, HSM (5/8)	7.4	52	1100	274	454	2000	No	1	N/A	N/A	PRF1	PRF1	NM_001083116.3:c.[673C>T];[673C>T]	2004 Protocol BMT	CR
22	78	78	M	1	No	Fever, HSM (7/8)	9	74	1400	710	585	13419	Yes	N/A	N/A	25628	PRF1	PRF1	NM_001083116.3:c.[1610A>C];[1610A>C]	2004 Protocol BMT	CR
23	106	111	F	1	Yes	Fever, HSM, CNS involvement (5/8)	6.5	85	400	203	236	29000	Yes	2	N/A	N/A	PRF1, STX, UNC13D, STXBP2	STXBP2	NM_006949.4:c.[1247-1G>C];[1247-1G>C]	HLH-94 BMT	CR
24	80	82	F	1	Yes	Fever, HSM (6/8)	6.2	39	700	353	130	1011	Yes	5	N/A	N/A	PRF1	PRF1	NM_001083116.3:c.[673C>T];[673C>T]	DEXA BMT	CR
25	2	2	M	1	Yes	Fever, HSM, Edema (8/8)	6.1	5	400	272	134	2036	Yes	1,5	0%	6684	PRF1	PRF1	NM_001083116.3:c.[673C>T];[673C>T]	DEXA BMT	deceased
26	12	81	M	1	No	Fever, HSM, Adenopathy, Elevated LFT, Rash, Edema (5/8)	6.3	19	0	345	129	555	N/A	4	N/A	N/A	NGS panel	STXBP2	NM_006949.4:c.[1247-1G>C];[1247-1G>C]	DEXA BMT	CR

27	48	54	M	3	No	Fever, HSM, Rash (genetic)	N/A	N/A	N/A	N/A	N/A	N/A	N/A	2,4	N/A	N/A	PRF1, STX, UNC13D, STXBP2	STXBP2	-	DEXA,IVIG BMT	CR
28	After birth	4	M	3	No	fever, rash (genetic)	N/A	N/A	N/A	N/A	N/A	N/A	N/A	2	N/A	N/A	PRF1, STX, UNC13D, STXBP2	STXBP2	-	2004 Protocol ATG,BMT	CR
29	96	99	M	1	Yes	Fever, CNS involvement (genetic)	8.6	71	2000	115	245	N/A	No	1	N/A	N/A	PRF1	PRF1	NM_001083116.3:c.[148G>A];[148G>A]	2004 Protocol BMT	CR
30	14	30	F	3	No	Fever, HSM, Rash, CNS involvement, Edema (6/8)	9.8	67	1900	350	123	3810	No	N/A	<20%	30936	PRF1, STX, UNC13D STXBP2	STXBP2	NM_006949.4:c.[474_483delinsGA];[326-30_326-23del]	2004 Protocol BMT	CR
31	1	2	F	4	Yes	Fever, HSM, Edema (genetic)	5.2	20	100	N/A	310	N/A	Yes	N/A	N/A	N/A	PRF1	PRF1	NM_001083116.3:c.[1122G>A];[1122G>A]	2004 Protocol BMT	CR
32	12	12	F	4	Yes	Fever, HSM, CNS involvement (7/8)	7.2	40	470	835	240	2179	Yes	N/A	N/A	16354	PRF1, UNC13D	UNC13D	NM_199242.3:c.[1389+1G>A];[1389+1G>A]	2004 Protocol BMT	deceased
33	1	1	M	4	Yes	Fever, HSM, Edema (6/8)	6.8	15	100	577	70	66,612	No	N/A	0%	N/A	NGS panel	XIAP	NM_001167.4(XIAP):c.[712C>T];[0]	2004 Protocol BMT	CR
34	2	0	M	1	Yes	Fever, HSM , Adenopathy Rash(6/8)	7.5	37	320	319	103	1270	Yes	N/A	N/A	N/A	PRF1, STX	STX11	-	2004 Protocol BMT	CR
35	2.5	2.5	M	1	Yes	Fever, HSM, Edema (7/8)	12	25	340	161	148	24,413	No	N/A	0%	9200	PRF1, STX, UNC13D	PRF1	NM_001083116.3:c.[1122G>A];[1122G>A]	2004 Protocol BMT	CR
36	0.75	0.75	M	4	No	Fever, HSM.CNS involvement (7/8)	7	18	180	780	80	1911	No	N/A	0%	7000	PRF1	PRF1	NM_001083116.3:c.[1122G>A];[1122G>A]	2004 Protocol BMT	CR
37	2	2	F	3	No	Fever, HSM, Rash, CNS involvement (7/8)	7.4	29	1060	696	86	14,199	No	N/A	0%	22,408	PRF1	PRF1	NM_001083116.3:c.[1122G>A];[10_11insCAGA]	2004 Protocol BMT	CR
38	12	14	M	1	Yes	Fever, HSM, Adenopathy, CNS involvement, Edema (7/8)	7.5	28	710	450	36	6912	No	5	0%	14,951	PRF1	PRF1	NM_001083116.3:c.[148G>A];[148G>A]	2004 Protocol ATG	deceased
39	2	3	F	1	N/A	Fever, HSM, CNS involvement (6/8)	8	2	0	415	N/A	29,256	No		N/A	2000	WES	N	-	2004 Protocol	deceased
40	3	9	F	1	Yes	Fever, HSM, CNS involvement (8/8)	7.3	41	480	373	70	700	Yes	3,4	8%	8000	WES	STXBP2	NM_006949.4:c.[1430C>T];[1430C>T]	2004 Protocol BMT	CR
41	1.5	4	F	1	Yes	Fever, HSM, CNS involvement, Edema (6/8)	5.7	16	400	593	133	1480	Yes	N/A	N/A	N/A	WES	STXBP2	NM_006949.4:c.[1430C>T];[1430C>T]	2004 Protocol BMT	CR
42	12	66	M	1	Yes	Fever, HSM, Adenopathy, CNS involvement (8/8)	6.4	10	170	1332	53	1546	Yes	N/A	<20%	11,998	WES	STXBP2	NM_006949.4:c.[1247-1G>C];[1247-1G>C]	2004 Protocol BMT	CR
43	10	11	M	1	Yes	Fever, HSM, Adenopathy (8/8)	7.1	21	110	1580	100	15,000	Yes	N/A	0%	3400	WES	PRF1	NM_001083116.3:c.[1122G>A];[1122G>A]	2004 Protocol BMT	CR
44	25	28	M	1	No	Fever, HSM, Adenopathy (6/8)	7.6	17	200	592	146	800	Yes	N/A	N/A	1064	WES	PRF1	NM_001083116.3:c.[148G>A];[148G>A]	2004 Protocol BMT	CR
45	4	4	M	1	Yes	Fever, HSM, CNS involvement (7/8)	7.5	15	70	460	75	10,606	Yes	N/A	0%	N/A	WES	PRF1	NM_001083116.3:c.[218_224del];[218_224del]	2004 Protocol BMT	CR
46	132	132	M	1	N/A	HSM, CNS involvement, Edema (genetic)	7.6	36	30	404	144	1267	No	N/A	N/A	N/A	WES	STXBP2	NM_006949.4:c.[1247-1G>C];[1247-1G>C]	2004 Protocol ATG	CR
47	120	132	M	1	Yes	Fever, HSM, CNS involvement, Edema (7/8)	7.1	52	1400	457	240	5266	Yes	N/A	N/A	4638	WES	STXBP2	NM_006949.4:c.[1247-1G>C];[1247-1G>C]	Dexa BMT	CR
48	1	3	M	1	Yes	Fever, HSM, CNS involvement, Edema (6/8)	6	8	170	N/A	140	5870	No	N/A	N/A	23,468	WES	PRF1	NM_001083116.3:c.[445G>A];[c.445G>A]	2004 Protocol BMT	N/A
49	N/A	18	F	1	Yes	N/A (genetic)	11	199	900	N/A	N/A	N/A	N/A	N/A	N/A	N/A	WES	STXBP2	NM_006949.4:c.[1247-1G>C];[1247-1G>C]	2004 Protocol BMT	CR
50	3	3	F	1	Yes	Fever, HSM, Rash, Edema (8/8)	8.4	30	700	766	N/A	20,000	Yes	N/A	N/A	N/A	N/D	-	-	2004 Protocol BMT	CR
51	0	72	M	1	Yes	Fever, HSM, Adenopathy, Edema (genetic)	N/A	N/A	100	700	123	40,000	N/A	N/A	N/A	N/A	WES	UNC13D	-	Dexa BMT	CR
52	9	120	M	1	Yes	Fever, HSM, Adenopathy (8/8)	7	16	0	452	816	809	Yes	1	2%	24,891	PRF1, STX, UNC13D, STXBP2, SH2D1A, XIAP	PRF1	NM_001083116.3:c.[673C>T];[673C>T]	2004 Protocol BMT	CR
53	3.5	3.5	F	1	Yes	Fever, CNS involvement (6/8)	6.6	32	600	609	116	2685	No	N/A	0%	14,103	UNC13D	UNC13D	NM_199242.3:c.[1754dup];[1754dup]	HLH-2004 BMT	deceased
54	13	14	M	1	Yes	Fever, CNS involvement (6/8)	8.6	40	500	230	122	260	Yes	N/A	5.7%	26,650	UNC13D	UNC13D	NM_199242.3:c.[1754dup];[1754dup]	HLH-94 BMT	deceased
55	10	12.5	F	3	No	Fever, HSM, CNS involvement, Edema (8/8)	7.4	37	1100	1172	149	1340	Yes	N/A	2.8%	11.289	PRF1, STX, UNC13D, STXBP2	STXBP2	NM_006949.4:c.[474_483delinsGA];[326-30_326-23del]	HLH-2004 BMT	CR
56	4	4.5	F	3	No	Fever, HSM, Edema (8/8)	4.9	20	1100	97	148	3000	Yes	N/A	3.7%	3723	PRF1, STX, UNC13D, STXBP2	PRF1	NM_001083116.3:c.[272C>T];[272C>T]	HLH-2004 BMT	CR
57	4	4	M	3	No	Fever, HSM (8/8)	7.3	17	300	378	119	514	Yes	N/A	0%	13,785	PRF1, STX, UNC13D, STXBP2	STXBP2	NM_006949.4:c.[326-30_326-23del];[326-30_326-23del]	HLH-2004 BMT	CR
58	1.5	2	M	4	Yes	Fever, HSM (7/8)	8.8	45	4100	320	32	24,654	No	1	0%	27,875	PRF1	PRF1	NM_001083116.3:c.[1122G>A];[1122G>A]	HLH-2004 BMT	CR

59	3.5	3.5	M	1	Yes	Fever, HSM, Rash, Edema (7/8)	8.6	14	8470	454	501	2949	Yes	N/A	0.5%	2149	N/D	-	-	HLH-2004 BMT	deceased
60	23.5	24	M	2	No	Fever, HSM, Rash (7/8)	7.6	21	100	192	170	1000	Yes	N/A	2.7%	25,000	PRF1, STX, UNC13D	UNC13D	NM_199242.3:c.[520C>T];[520C>T]	HLH-2004 BMT	CR
61	69.5	74	M	1	No	Fever, HSM (8/8)	7.8	49	700	481	150	1565	Yes	2	6.8%	12,558	PRF1, STX, UNC13D, STXBP2	STXBP2	NM_006949.4:c.[1247-1G>C];[1247-1G>C]	HLH-2004 BMT	deceased
62	14	19	F	1	Yes	Fever, HSM, Rash (7/8)	7.7	41	1000	194	332	1597	Yes	N/A	0%	10,159	UNC13D	UNC13D	NM_199242.3:c.[1754dup];[1754dup]	Dexa BMT	CR
63	0.75	1	M	4	No	Fever, HSM, Elevated LFT, Rash, Edema (7/8)	8.1	27	800	1044	75	11,773	Yes	1,5	N/A	12,000	PRF1	PRF1	NM_001083116.3:c.[1122G>A];[1122G>A]	HLH-94 BMT	CR
64	5	6	M	3	No	Fever, HSM (7/8)	9.4	34	500	946	130	3621	No	4	10%	10,440	NGS panel	STXBP2	NM_006949.4:c.[326-30_326-23del];[326-30_326-23del]	HLH-94 BMT	CR
65	2	5	M	5	No	Fever (6/8)	9.2	91	400	257	77	1055	No	2,4	3.7%	9558	UNC13D	UNC13D	NM_199242.3:c.[1240_1241insAGCCACTTCGGCC];[1240_1241insAGCCACTTCGGCC]	HLH-94 BMT	deceased
66	0	1.5	M	2	No	Only cytopenia (genetic)	10.5	13	200	154	100	13,000	No	3	N/A	8131	NGS panel	UNC13D	NM_199242.3:c.[1663_1664delAG];[743T>C]	HLH-94 Emapulumab BMT	CR
67	12	12	M	1	Yes	CNS involvement (genetic)	7.9	111	700	268	83	863	N/A	N/A	N/A	N/A	PRF1	PRF1	NM_001083116.3:c.[673C>T];[673C>T]	HLH-2004 BMT	N/A
68	2	2	M	1	Yes	Fever, HSM (genetic)	N/A	N/A	N/A	N/A	N/A	N/A	N/A	N/A	N/A	N/A	PRF1, STX, UNC13D, STXBP2	PRF1<br>UNC13D	NM_001083116.3:c.[272C>T];[272C>T]<br>NM_199242.3:c.[2341G>A];[3145C>G]	HLH-2004	deceased
69	21	21	F	1	N/A	Fever, HSM, Rash, Adenopathy, Edema (7/8)	6	2	100	610	430	25,000	No	N/A	<20%	10,000	PRF1, STX, UNC13D, STXBP2	N	-	Dexa	deceased
70	0.75	6	F	1	N/A	Fever, HSM, CNS involvement (6/8)	7.5	6	600	768	30	16,800	No	N/A	N	11,170	WES	N	-	HLH-2004	N/A
71	10	15	F	3	No	Fever, Elevated LFT, Edema (6/8)	7.3	32	1200	740	85	30,000	Yes	N/A	N	10,800	PRF1, STX, UNC13D	N	-	HLH-2004 BMT	CR
72	156	156	F	2	No	Fever, Adenopathy (7/8)	9.1	280	0	1000	83	845	Yes	N/A	0.5%	8361	PRF1, STX, UNC13D, STXBP2	N	-	2004 Protocol Alemtuzumab	CR
73	33	33	F	1	Yes	Fever, HSM (5/8)	11.2	20	0	N/A	N/A	24,518	N/A	N/A	N/A	6081	NGS panel	XIAP	NM_001167.4(XIAP):c.[712C>T];[0]	HLH-2004 BMT	deceased
74	15	15	M	3	No	Fever, HSM, Edema (6/8)	7.5	30	500	403	250	5669	No	N/A	N	9000	PRF1, STX, UNC13D, STXBP2,XIAP,SH2DA1	N	-	HLH-2004 BMT	CR
75	58	58	F	3	No	Fever, HSM, Edema, EBV+ (8/8)	10.1	25	800	461	80	8317	Yes	N/A	2.8%	12,000	PRF1, STX, UNC13D, STXBP2	N	-	HLH-2004 Mabthera Alemtuzumab	deceased
76	108	108	M	3	N/A	Fever, Adenopathy (6/8)	10.1	124	800	274	80	3137	Yes	N/A	9.3%	10,567	PRF1, STX, UNC13D, STXBP2, SH2D1A,XIAP	N	-	HLH-2004	CR
77	21	21	F	3	No	Fever, Rash, CNS involvement (6/8)	11.3	43	830	601	126	8640	No	N/A	0%	5317	WES	N	-	HLH-2004	CR
78	25	25	M	2	No	Fever, CNS involvement, EBV+ (6/8)	9.3	33	900	737	32	54,158	No	1	9.5%	12,181	PRF1, STX, UNC13D, STXBP2, SH2D1A,XIAP	N	-	HLH-2004 Mabthera	CR
79	126	126	F	2/3	No	Fever, HSM, Adenopathy, EBV+ (5/8)	10.5	135	1700	106	139	7550	No	N/A	N	6940	PRF1, STX, UNC13D, STXBP2, ITK, STX, CD27	N	-	HLH-2004 Mabthera	CR
80	36	37	F	2	No	Fever, HSM, Rash, Elevated LFT (5/8)	8.1	82	600	229	338	344	Yes	1	N	6090	NGS panel	N	-	HLH-94 BMT	CR
81	3	4	M	3	No	Fever, Elevated LFT (5/8)	11	80	300	N/A	N/A	50,000	Yes	1,4	N	6242	NGS panel	N	-	HLH-94	CR
82	120	120	F	2	No	Fever, HSM (6/8)	9.4	56	800	979	146	7450	No	N/A	N	4916	PRF1, UNC13D, STXBP2, ITK	N	-	HLH-2004	CR
83	72	72	F	3	No	Fever, HSM, EBV+ (7/8)	8	49	800	221	74	1179	Yes	N/A	N	12,839	NGS panel	N	-	HLH-2004 Mabthera	CR
84	132	132	F	2	No	Fever, Adenopathy, EBV+ (5/8)	9	4	1400	243	177	1898	Yes	N/A	N	2906	PRF1, STX, UNC13D, STXBP2	N	-	HLH-2004 Mabthera	CR
85	22	23	F	2/3	No	Fever, HSM, Adenopathy (5/8)	7.8	121	700	540	142	356	No	N/A	N/A	12,459	PRF1, STX, UNC13D, STXBP2	N	-	Dexa	CR
86	60	60	F	3	No	Fever, HSM (5/8)	10.3	471	4900	190	120	680	Yes	N/A	N/A	2137	N/D	-	-	Dexa	CR
87	8	9	M	1	No	Fever, HSM, Rash (6/8)	8.7	8	700	330	89	56,796	No	N/A	N	12,459	N/D	-	-	HLH-2004	CR

88	168	168	M	3	No	HSM (5/8)	10.4	100	400	230	150	3263	Yes	N/A	N	8090	PRF1, UNC13D	N	-	Dexa,IVIG	CR
89	N/A	144	F	1	Yes	Fever, HSM, Adenopathy (5/8)	6.5	6	700	N/A	N/A	>500	Yes	N/A	N/A	N/A	N/D	-	-	HLH-94	deceased
90	62	66	M	3	No	Fever, HSM, Adenopathy, Edema (7/8)	9.1	9	100	800	508	30,000	N/A	N/A	3%	3700	N/D	-	-	HLH-2004	deceased
91	116	116	M	1	N/A	Fever, HSM, Adenopathy, Edema (7/8)	5.7	40	0	818	136	12,705	No	N/A	N/A	N/A	N/D	-	-	Dexa	CR
92	0	2	F	3	No	Only cytopenia (6/8)	8.7	5	500	555	113	4872	Yes	N/A	N/A	6572	N/D	-	-	HLH-2004	CR
93	4	4	M	1	No	Fever, HSM, Rash, Adenopathy, Edema, CNS involvement (5/8)	6.6	25	170	N/A	N/A	>500	Yes	N/A	N/A	N/A	N/D	-	-	HLH-2004 BMT	CR
94	1	1	F	2	N/A	Fever, HSM, Edema (5/8)	9	11	1200	N/A	N/A	>500	Yes	N/A	N/A	N/A	N/D	-	-	HLH-2004	CR
95	7	7	F	3	No	Fever, HSM (5/8)	5.2	80	700	N/A	N/A	1900	Yes	N/A	N/A	N/A	N/D	-	-	HLH-94	N/A
96	14	15	F	3	No	Fever, HSM, Rash (5/8)	7.9	49	1700	N/A	N/A	>500	Yes	N/A	N/A	N/A	N/D	-	-	HLH-94 BMT	CR
97	204	205	F	2	No	Fever, HSM (6/8)	8.5	215	0	643	100	997	No	N/A	N/A	6152	N/D	-	--	HLH-2004	deceased
98	145	145	F	3	No	Fever, HSM, Rash (6/8)	9.7	278	2000	330	120	6900	Yes	N/A	3,4	3509	N/D	-	-	Dexa	CR

Table S2. Patient's characteristics.

Legend: HLH- Hemophagocytic lymphohistiocytosis; LFT- liver function test; Hb- Hemoglobin; PLT -platelet; ANC- absolute neutrophil count; TG-triglycerides; Fib.-fibrinogen; NK- natural killer; CR- complete remission; N-normal; N/A -not available; N/D-not done; NGS- next generation sequencing; WES- whole exom sequencing; Tx -treatment. Ethnicity: Arab Muslim=1, Sephardic Jew=2, Ashkenazi Jew=3, Druze=4, Ethiopian Jew=5, Flow cytometry: CD107a normal =1, CD107a low=2, CD107a absent=3, perforin normal=4, perforin abnormal=5

Table S1- HLH-2004 Diagnostic Criteria

A. Molecular diagnosis consistent with HLH

OR

B. 5/8 diagnostic criteria
    1. Clinical criteria
        - Fever
        - Splenomegaly
    2. Laboratory criteria
        - Cytopenias ($\geq$ 2/3 lineages)
        - Hypertriglyceridemia +/or Hypofibrinogenemia
    3. Histopathological criteria
        - Hemophagocytosis in BM, spleen, l.n. (no malignancy)
    4. New criteria
        - Low/absent NK activity
        - Ferritin > 500 mcg/l
        - Soluble IL-2 Receptor (CD25) > 2400 U/ml

Henter et al, *Pediatr Blood Cancer 2007*

Table S3 NGS Panel gene list 2025

Gene List_S3491792:

ABCB6, ABCB7, ABCG5, ABCG8, ACD, ACKR1 promoter (rs2814778), ACO1, ACTB, ACTN1, ADA, ADAMTS13, ADAR, ADH5, ADRA2A, AICDA, AIRE, AK1, AK2, ALAS2, ALDH2, ALDOA, AMN, ANK1, ANKRD26 (including 5'UTR), ANO6, AP3B1, AP3D1, APAF1, DCLRE1B (APOLLO/SNM1B), ARHGEF4, ARPC1B, ASXL1, ATM, ATP11C, ATP7B, ATR, ATRX, B2M, BCL2L11, BCORL1, BHLHE41, BLM, BLOC1S3, BLOC1S6, BPGM, BRCA1, BRCA2, BRIP1, BTK, C15ORF41, C6ORF25 (MPIG6B), CAD, CARD11, CARD9, CASP10, CASP8, CBL, CBLIF, CD19, CD247, CD27, CD36, CD3D, CD3E, CD3G, CD40, CD40LG, CD59, CD70, CDAN1, CDC42, CDKN1B, CDKN2A, CEBPA, CECR1 (ADA2), CHEK2, CIITA, CLCN7, CLPB, COPZ1, CORO1A, COX4I1, COX4I2, CP, CPO, CSF3R, CSMD1, CTC1, CTLA4, CTPS1, CUBN, CXCR2, CXCR4, CYB5R3, CYCS, DCLRE1C, DDX41, DHFR, DHX38, DIAPH1, DKC1, DNAJC21, DNASE2, DNMT3A, DOCK2, DOCK8, DPP9, DTNBP1, DUT, EED, EFL1, EGLN1, EGLN2, EGLN3, EIF2AK3, ELANE, ENOSF1, EPAS1, EPB41, EPB42, EPO, EPOR, ERCC1, ERCC4, ERCC6L2, ETV6, F2R, F2RL3, FAAP100, FAAP24, FADD, FANCA, FANCB, FANCC, FANCD2, FANCE, FANCF, FANCG, FANCI, FANCL, FANCM, FAS, FASLG, FECH, FERMT3, FLI1, FLNA, FLNB, FLT3, FLVCR1, FLVCR2, FOXP3, FTL, FXN, FYB, G6PC3, G6PD, GALE, GAR1, GATA1, GATA2, GBA, GCLC, GFI1, GFI1B, GINS1, GLRX5, GNE, GNL3, GP1BA, GP1BB, GP6, GP9, GPI, GPX1, GRHL2, GSR, GSS, HAMP, HAVCR2 (TIM3), HAX1, HEATR3, HFE, HIF1A, HIF1AN, HIF3A, HJV, HK1, HOXA11, HPS1, HPS3, HPS4, HPS5, HPS6, HSCB, HSPA9, HYOU1, ICOS, IDH1, IDH2, IKBKG, IKZF1, IKZF2, IL10, IL10RA, IL10RB, IL12B, IL12RB1, IL21, IL23A, IL2RA, IL2RG, IL7R, INO80, IRF8, ISG15, ITCH, ITGA2, ITGA2B, ITGB3, ITK, ITPKC, ITPR3, JAGN1, JAK1, JAK2, JAK3, JARID2, KCNN4, KDM6A, KIF23, KIT, KLF1, KMT2A, KRAS, LAMTOR2, LARS2, LAT, LCK, LCP1, LIG4, LPIN2, LRBA, LRRC3, LYST, LZTR1, MAD2L2, MADD, MAGT1, MAP2K1, MAPK1, MASTL, MBD4, MCM10, MCM2, MCM4, MDC1, MECOM, MEIS1, MET, MITF, MKL1, MOGS, MPL, MRAS, MRE11A, MS4A1, MST1, MT-ATP6, MTRR, MVK, MYH9, MYSM1, NAF1, NBAS, NBEA, NBEAL2, NBN, NDUFB11, NF1, NFATC1, NFKB1, NFKB2, NFKBIA, NHEJ1, NHP2, NLRC4, NM_001861 (COX4I1), NOP10, NPM1, NRAS, NSD1, NSMCE3, NT5C3A, NUF2, ORAI1, OS9, P2RY12, PALB2, PARN, PARP4, PAX5, PGK1, PGM3, PIEZO1, PIGA, PIK3CD, PIK3R1, PKLR, PLA2G4A, PLAU, PLCG2, POT1, PPOX, PRDX2, PRF1, PRKACG, PRKCD, PRKDC, PTGS1, PTPN11, PTPRC, PTPRJ, PUS1, RAB27A, RAC2, RACGAP1, RAD50, RAD51, RAD51C, RAF1, RAG1, RAP1A, RASA2, RASGRP1, RASGRP2, RBM8A, RBSN, RC3H1, RFWD3, RFX5, RFXANK, RFXAP, RHAG, RHOG, RIPK2, RIT1, RMRP, RNF168, RPA1, RPL11, RPL15, RPL18, RPL26, RPL27, RPL31, RPL35, RPL35A, RPL5, RPL8, RPL9, RPS10, RPS14, RPS15A, RPS17, RPS18, RPS19, RPS20, RPS24, RPS26, RPS27, RPS28, RPS29, RPS7, RPS8, RRAS, RRAS2, RREB1, RTEL1, RUNX1, SAMD9, SAMD9L, SBDS, SEC23B, SEC61A1, SEMA7A, SENP7, SETBP1, SF1, SH2B3, SH2D1A, SHOC2, SLC11A2, SLC19A2, SLC25A38, SLC29A1, SLC2A1, SLC30A10, SLC37A4, SLC40A1, SLC4A1, SLC7A7, SLFN14, SLX4, SMARCD2, SOCS1, SOS1, SOS2, SPTA1, SPTB, SRC, SRP54, SRP68, SRP72, SRSF2, STAT1, STAT3, STAT5B,

STEAP3, STIM1, STK4, STN1, STX11, STXBP2, SUPT5H, TAP1, TAP2, TAPBP, TAZ, TBXA2R, TBXAS1, TCIRG1, TCN2, TERC, TERF1, TERF2, TERT, TET2, TF, TFR2, TFRC, THBD, THPO, TINF2, TLR8, TMPRSS6, TNFRSF13B, TNFRSF25, TNFSF13B, TP53, TPI1, TPM4, TPP2, TRAF3, TRNT1, TSR2, TTC7A, TUBB, TUBB1, TYK2, TYMS, U2AF1, U2AF2, UBE2T, UGT1A1, UNC13D, UNG, UROS, USB1, VHL, VIPAS39, VPS13B, VPS33B, VPS45, VPS4A, VWF, WAS, WDR1, WIPF1, WRAP53, WRN, WT1, XIAP, XRCC2, YARS2, ZAP70, ZCCHC8, ZNF197, ZNFX1, ZRSR2, chr6:27495814-27495895, chr3:128202000-128202300 (GATA2 intron 4), chr19:48965100-48967000 (FTL promoter IRE), chr19:7705695-7705785 (STXBP2 intron 5).
